# Huffing and Puffing: A Rare Case of Tracheal Adenoid Cystic Carcinoma

**DOI:** 10.7759/cureus.11887

**Published:** 2020-12-03

**Authors:** Sarbjot Grewal, Namitha Malakkla, Jasleen Kaur, Pedram Ansari, Ravi Rao

**Affiliations:** 1 Internal Medicine, Saint Agnes Medical Center, Fresno, USA; 2 Hematology and Oncology, Saint Agnes Medical Center, Fresno, USA

**Keywords:** tracheal adenoid cystic carcinoma, neoplasms of trachea, refractory asthma

## Abstract

We present a case of a 78-year-old woman presenting with dyspnea and discovered to have an intra-tracheal tumor. Resection revealed this to be an adenoid cystic carcinoma (ACC) of the trachea, which is a very rare entity. She was treated with subtotal resection followed by radiation to the residual tumor, following which she relapsed six years later. Due to its indolent nature, she continues to be followed without any therapy. The biology, clinical features, and natural history of this rare tumor will be discussed.

## Introduction

Primary tracheal tumors, especially adenoid cystic carcinoma, are rare entities. Most tumors that involve the trachea originate either in the lung, directly infiltrate from adjacent organs, or are metastases from elsewhere. Adenoid cystic carcinoma (ACC) is a histology that is most often found to originate from the major salivary glands, but rarely has been noted to arise in the mucus glands in the trachea. These tumors often cause obstructive symptoms and may often mimic asthma-like symptoms including dyspnea and wheezing. Herein we discuss the case of a patient who presented with dyspnea and was subsequently discovered to have a primary tracheal adenoid cystic carcinoma.

## Case presentation

**Figure 1:**
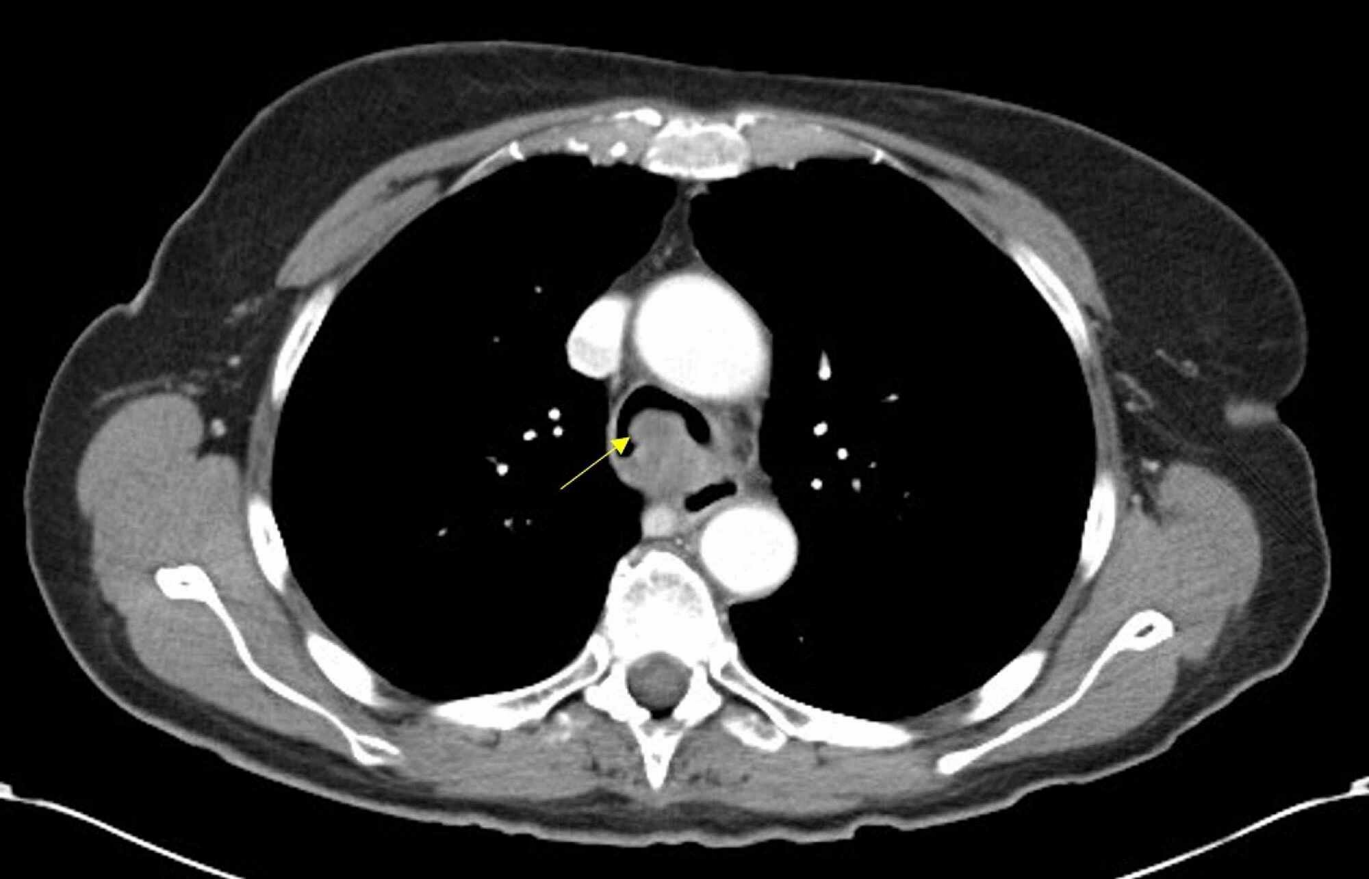
Axial cut of CT Chest showing luminal narrowing due to tracheal mass.

**Figure 2:**
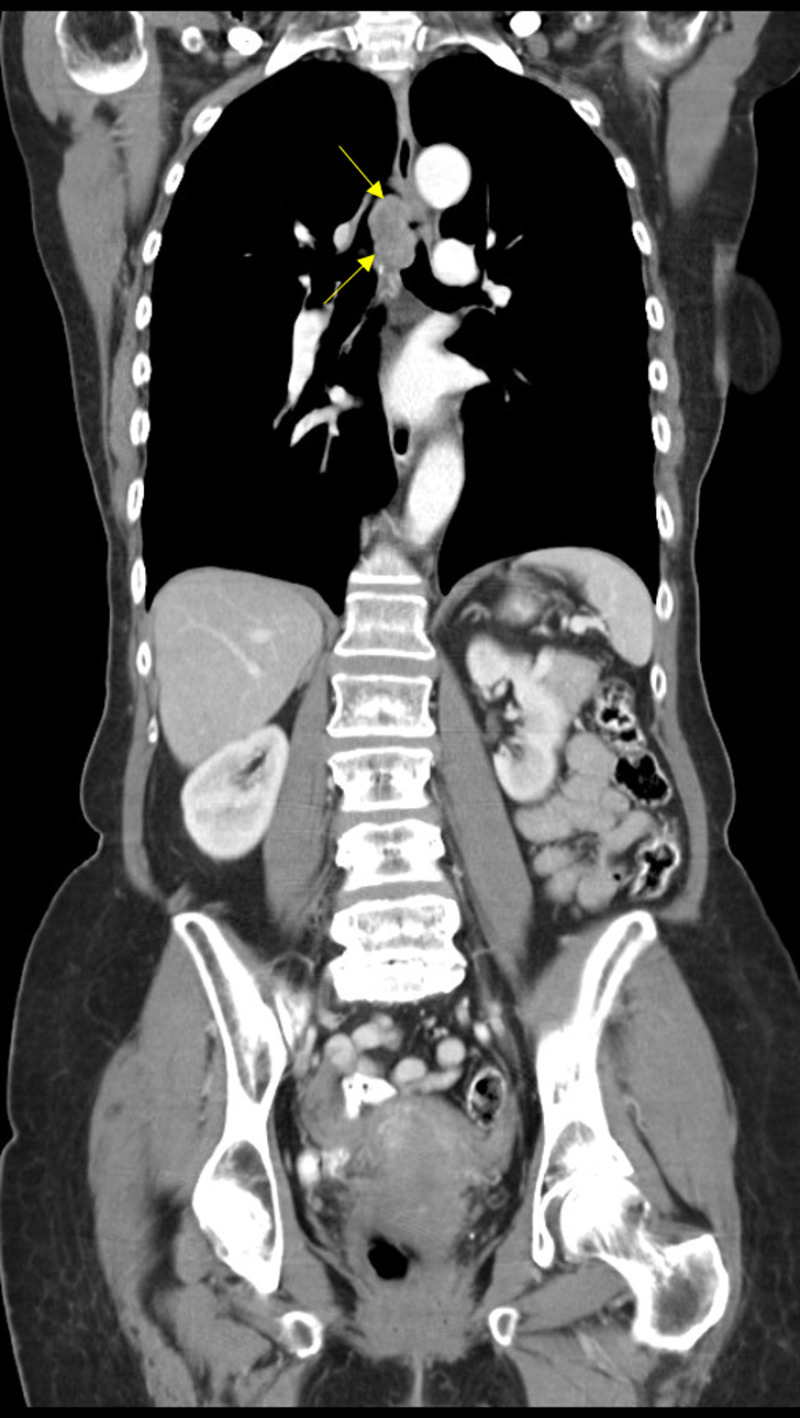
Coronal view of CT Chest showing a 4 cm mass near the trachea.

A 78-year-old Caucasian female with no smoking history presented to her primary care physician with cough, dyspnea, and wheezing for a few months. Pulmonary function testing was indicative of upper airway obstructive disease. She was diagnosed with adult-onset asthma and treated with bronchodilator therapy. Her symptoms did not respond appropriately to bronchodilator therapy and within the next few months she developed dysphagia. She then had chest CT with contrast that revealed a 4 cm tumor that seemed to arise in the posterior trachea and caused partial obstruction of the tracheal lumen (Figure 1, 2).

**Figure 3:**
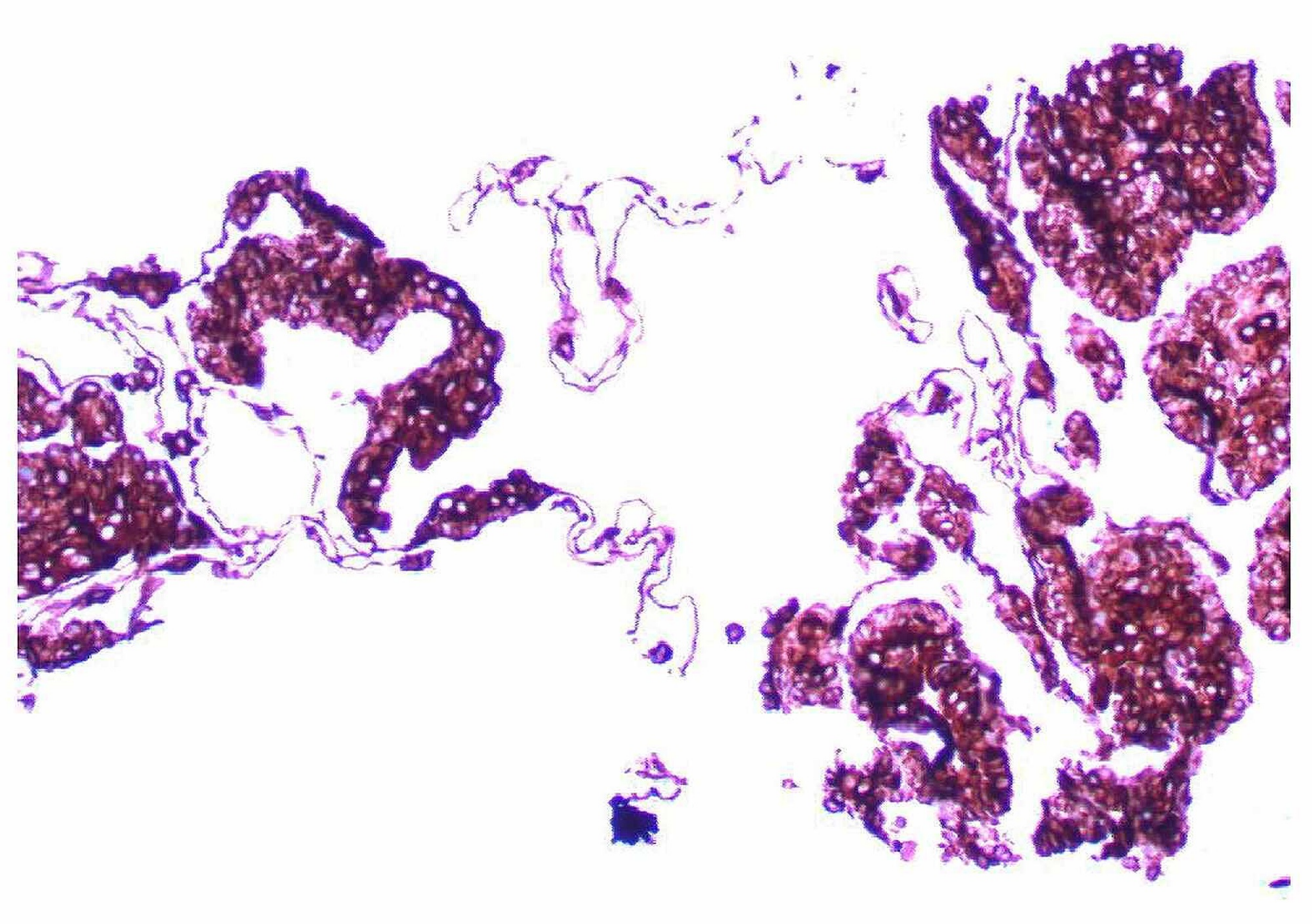
Right lower lobe lung nodule biopsy: Adenoid cystic carcinoma. Immunohistochemistry (IHC) CK7+, 200x.

**Figure 4:**
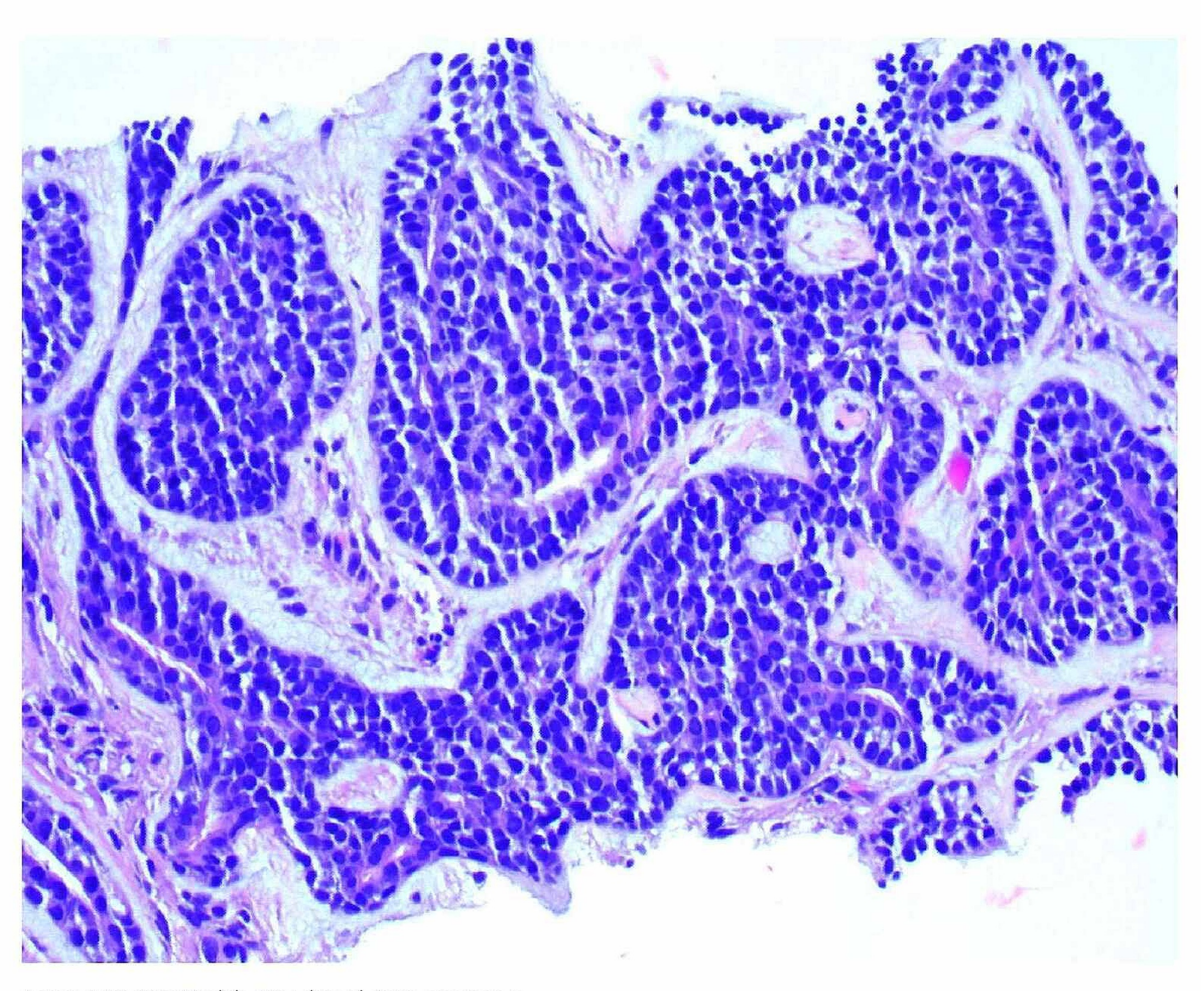
Right lower lobe lung nodule biopsy: Adenoid cystic carcinoma.

Rigid bronchoscopy was performed and 90% of the mass was resected, as estimated by the operating surgeon. Pathology revealed adenoid cystic carcinoma. Repeat positron emission tomography (PET) scan did not reveal any other sites of disease; the trachea was confirmed to be the site of the primary. Her symptoms completely resolved after resection. However, since there was residual intramural disease in the trachea that could not be resected, she was treated with radiation with curative intent. The patient was then placed on a protocol of serial observation with periodic imaging. After six years, the patient developed biopsy-proven lung metastases as shown in Figure 3 and Figure 4. She was minimally symptomatic, so is being expectantly followed and no further interventions or treatment were deemed necessary. Her functional status declined due to concurrent worsening of Parkinson's disease. Due to the diffuse nature of her disease, surgery and radiation were not appropriate. Next-generation sequencing did not reveal any actionable mutations, ruling out the use of any targeted therapy. Two years after having the recurrence she remains under observation.

## Discussion

Tumors that involve the upper airway are rare occurrences [1]. Most tumors in this location are benign, while other malignant tumors involving the trachea by direct infiltration (eg, from the esophagus), with primary malignant tumors being exceedingly rare. The incidence of primary tracheal tumors is less than 0.2 per 100,000 persons per year. The most common malignant neoplasms of the trachea are squamous cell carcinoma (SCC). ACC, which normally arises in the salivary glands in the head and neck, rarely arises in the mucosal glands of the trachea [2]. Due to the rarity of this tumor, much of the information on this tumor is obtained from case reports.

ACCs typically present at 40 to 50 years of age and seem to occur in both sexes equally. There is increased risk in smokers. Because of the confounding symptoms, the typical time to make the diagnosis from the onset of symptoms is one year. Since they present with symptoms of airway obstruction, they are usually initially diagnosed as asthma or chronic obstructive pulmonary disease (COPD).

A multidisciplinary team approach, including medical, surgical, and radiation oncology specialists, should be employed for optimal results. Data on optimal management strategy is limited due to the rarity of the disease. Surgical resection is the mainstay of therapy, with data from case series suggesting that extent of resection corresponds to survival [3-4]. Surgical resection with end-to-end anastomosis has shown promising results. Postoperative radiation is recommended for patients with unresectable tumors or who undergo incomplete resection with positive margins. A study measuring long-term survival in patients with complete versus incomplete resection showed five-year survival rates of 82% versus 77% respectively, suggesting that complete resection with negative tumor margins has a significantly better outcome [4-5]. There is little data on the use of chemotherapy in tracheal ACC and the results of prior studies have been poor; thus, there is a minimal role for its use especially in resectable tumors. In some cases, bronchoscopy was also performed at six and 12 months after radiation therapy, confirming disappearance of the tumor mass [5]. 

If the tumor is unresectable or partially resectable with positive margins, primary or postoperative radiation therapy is recommended [6]. Surveillance with periodic imaging and bronchoscopy may have a role in survivors. In this case, due to the extent of infiltration into the carina, the tumor was partially resectable and required subtotal resection of the trachea. Following resection, the patient underwent postoperative radiation therapy and continues to live a healthy lifestyle with complications due to Parkinson's disease nine years following her final treatment.

## Conclusions

Primary malignant tumors of the airway are rare, and pose a diagnostic challenge as they often mimic benign disease of the airway. We present a case of partially obstructive malignant tracheal ACC, where the patient presented with symptoms suggestive of asthma. Primary tracheal ACC can be a difficult diagnosis to make, given the misleading presentation. Smoking does have a correlation as mentioned earlier but our patient did not have a history of smoking. It is vital for all physicians to consider complex malignant processes in the setting of obstructive lung disease if a patient is refractory to standard treatment. Early multidisciplinary management of these patients often leads to dramatic improvement in long-term survival. 
